# Aligned Ice Templated Biomaterial Strategies for the Musculoskeletal System

**DOI:** 10.1002/adhm.202203205

**Published:** 2023-05-01

**Authors:** Florencia Diaz, Nicholas Forsyth, Aldo R. Boccaccini

**Affiliations:** ^1^ Department of Materials Science and Engineering Institute of Biomaterials University of Erlangen‐Nuremberg 91058 Erlangen Germany; ^2^ The Guy Hilton Research Laboratories School of Pharmacy and Bioengineering Faculty of Medicine and Health Sciences Keele University Stoke on Trent ST4 7QB UK

**Keywords:** aligned scaffolds, biomaterials, bone tissue engineering, musculoskeletal system, unidirectional freezing

## Abstract

Aligned pore structures present many advantages when conceiving biomaterial strategies for treatment of musculoskeletal disorders. Aligned ice templating (AIT) is one of the many different techniques capable of producing anisotropic porous scaffolds; its high versatility allows for the formation of structures with tunable pore sizes, as well as the use of many different materials. AIT has been found to yield improved compressive properties for bone tissue engineering (BTE), as well as higher tensile strength and optimized cellular alignment and proliferation in tendon and muscle repair applications. This review evaluates the work that has been done in the last decade toward the production of aligned pore structures by AIT with an outlook on the musculoskeletal system. This work describes the fundamentals of the AIT technique and focuses on the research carried out to optimize the biomechanical properties of scaffolds by modifying the pore structure, categorizing by material type and application. Related topics including growth factor incorporation into AIT scaffolds, drug delivery applications, and studies about immune system response will be discussed.

## Graphical abstract

This review critically examines the existing literature regarding aligned ice templated scaffolds for applications in musculoskeletal tissue engineering. This work describes the fundamentals of this technique and focuses on the research carried out to optimize the biomechanical properties of scaffolds by modifying the pore structure, categorizing by material type, and application. Areas requiring further research are identified.

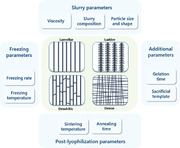

## 1 Introduction

Unidirectional ice templating is a reliable and highly reproducible technique used in diverse fields to produce aligned pore structures in a wide array of materials, ranging from polymers to ceramics, metals and composites.^[^ ^1^, ^2^ ^]^ It has attracted interest from areas including energy production,^[^ ^3^ ^]^ considering that energy production systems feature a porous element that provides mechanical support and conductivity, introducing pore alignment could improve the fluid permeation in these devices;^[^ ^4^ ^]^ catalysis, as it could produce three dimensional structures with a lower cost;^[^ ^5^ ^]^ and three‐dimensional cell culture.^[^ ^6^ ^]^

Its versatility has made it an attractive option to produce biomaterials with aligned macropores in the range of 1−400 µm, allowing for multiple applications in, among others, myocardial, bone, skin, neural, and connective tissue engineering.^[^ ^7^, ^8^, ^9^ ^]^

**Figure 1:**
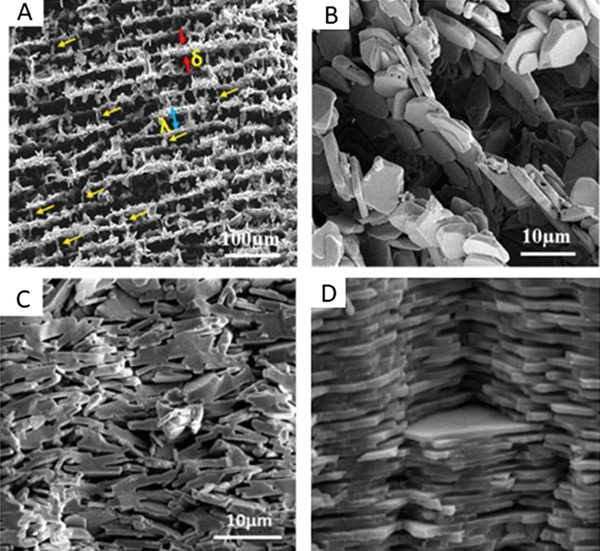
Mimicking of natural nacre structure via aligned ice templating (AIT). a) Scanning electron microscopy (SEM) image of sintered alumina platelet scaffold with yellow arrows highlighting the alignment. b) SEM image at higher magnification exhibits the arrangement of the alumina platelets. c) SEM image of the scaffold after compression, exhibiting the reduction in the pore size. d) SEM image of natural nacre. (a–c) Reproduced with permission.^[^ ^14^ ^]^ Copyright 2021, Elsevier.

Though the method to achieve aligned ice templated materials has been known and investigated since the 1980s,^[^ ^10^ ^]^ the technique re‐emerged in the mid‐2000s.^[^ ^11^ ^]^ This resurgence could be attributed to growing interest in the development of “green” techniques, with low environmental impact; as well as the need for a technology to imitate three‐dimensional natural materials such as bone and nacre.^[^ ^12^ ^]^ Nacre is a widely available natural material composed of lamina of aragonite platelets; its high strength and toughness have attracted interest from the scientific community for decades. Layer‐by‐layer assembly and electrodeposition techniques have been used in attempting to recreate its structure, with limited success, as they can only produce two‐dimensional materials.^[^ ^13^ ^]^ On the other hand, using alumina platelets and ice templating, aligned three dimensional structures could be achieved, which mimic the natural shape of nacre (**Figure** **1**). Terms such as “freeze casting”, “ice templating”, “ice segregation induced self‐assembly”, and “unidirectional freezing” are used interchangeably to refer to this technique.^[^ ^15^ ^]^

In the case of the musculoskeletal system, aligned ice templating (AIT) has emerged as an alternative to other commonly used techniques, such as 3D printing and electrospinning, in hopes to remedy some of their limitations. In the case of the former, it can be challenging to obtain the right mechanical properties required for “harder” materials, such as those biomaterials relevant for bone and tendon regeneration. For the latter, there have been concerns regarding cell infiltration, due to the nanometric dimensions of the pores created between the fibers. Ice templating, though not capable of generating the nanometric fibers produced by electrospinning or the precise features of 3D printed scaffolds, is a simple process not requiring complex equipment and can generate oriented macropores in the appropriate range for many applications.

The musculoskeletal system, understood to comprise the tissues involved in locomotion, shape and stability of the body, often presents an architecture that can be mimicked by aligned freeze casted structures. Tissues including muscle, tendon, and ligament present an aligned, fibrous structure,^[^ ^16^, ^17^ ^]^ while the majority of bone tissue in humans is made up of lamellae of hydroxyapatite (HA) and collagen.^[^ ^18^ ^]^

It is important to note that musculoskeletal disorders place a significant burden on the healthcare system. Between 5.4% and 12.3% of total health expenses in developed countries can be attributed to this group of diseases, which include lower back pain, osteoarthritis, neck pain, and many more.^[^ ^19^ ^]^ A study has indicated that musculoskeletal injuries and diseases represent a major cause of lost work hours;^[^ ^20^ ^]^ while up to 38% of occupational diseases are related to the musculoskeletal system and can cost the European Union between 0.5% and 2% of its gross domestic product.^[^ ^20^ ^]^ In a report issued in 2019, the European Risk Observatory has remarked that prevention and improved treatment of these diseases should be a priority, and highlighted a need in increasing the knowledge and existing policy EU‐wide.^[^ ^21^ ^]^

To obtain an aligned ice templated material, a (typically aqueous) solution is put in contact with a cold surface, at temperatures lower than the solvents freezing point.^[^ ^22^ ^]^ This triggers the nucleation of ice in supercooled water, followed by the growth of the ice nuclei and the segregation of the solute into a template, caused by a stark reduction of the solubility of the solute in ice.^[^ ^23^ ^]^ Ice functions as a porogen, which is removed via lyophilization or thawing, resulting in a pore structure that will be highly anisotropic and aligned.^[^ ^24^ ^]^

This microstructure can have similarities with the native tissues, bringing with it many advantages. An almost 4× increase in ultimate tensile strength has been measured for aligned ice templated porous keratin scaffolds compared to their isotropic versions;^[^ ^25^ ^]^ whereas similar results were found for the ultimate compressive strength of scaffolds produced from a gelatin and chitosan blend.^[^ ^26^ ^]^ Caliari et al.^[^ ^27^ ^]^ found a significant increase in tenocyte alignment and proliferation on an anisotropic collagen‐glycosaminoglycan scaffold, regardless of pore size, compared to a randomly oriented scaffold. Furthermore, osteogenic potential can improve in the presence of aligned pores, leading to more mature osteocytes and increased calcium deposition on anisotropic collagen scaffolds.^[^ ^28^ ^]^ Pore alignment is also known to encourage macrophage differentiation to the M2 anti‐inflammatory phenotype, especially important for certain tissues such as tendon, which often suffer excessive scarring and poor healing capabilities.^[^ ^29^ ^]^ This technique has drawn significant interest as of late, with multiple review papers detailing the physics and mechanics of ice templating of different materials published in recent years.^[^ ^22^, ^30^ ^,31]^

In this review, we will focus on and discuss potential applications of unidirectional freezing for developing scaffolds for different tissues in the musculoskeletal system, providing a thorough summary of the biomaterials currently and formerly under investigation, and their properties. This review is organized as follows: Section 2 introduces and summarizes the basis of the AIT technique. Section 3 describes the different materials used in AIT for bone tissue engineering (BTE), including ceramics, polymers, and metals. Sections 4, 5, 6 provide a summary on the research on AIT applications for tendon, skeletal muscle, and cartilage applications, respectively. Finally, in Section 7, the opportunities and challenges of AIT strategies for biomaterials for the musculoskeletal system are discussed.

## 2 Basic Principles of Ice Templating

**Figure 2:**
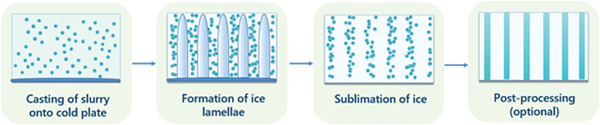
Schematic diagram showing the different stages of the ice templating process. The morphology of the ice crystals, and therefore the final pore structure, will be influenced by the process parameters, including freezing temperature, slurry composition, and more.

The process takes place in several steps (**Figure** **2**). First, a solution or slurry is prepared, containing the material to be templated, the solvent, and any additives such as binders or dispersants. This is then cast onto a mold, which on one end contacts the cooled surface and the other end is either exposed to room temperature or insulated, and can be connected to a temperature controller. Often, this mold is insulated all throughout its sides, particularly if using liquid nitrogen as a freezing agent, to prevent lateral ice formation. This temperature gradient allows for the formation of a freezing front, yielding aligned ice lamellae. On the first step of the freezing process, an ice layer is formed quickly on the side of the mold in contact with the cold surface, subsequently, ice lamellae start to grow in the direction of the temperature gradient. Once fully frozen, the sample is either dried under vacuum, to sublimate the ice particles, or thawed, resulting in a porous scaffold, also known as a template. The structure reveals a dense zone on the bottom of the scaffold, caused by the initial fast freezing stage that creates the first layer of ice very rapidly, and thus entrapment of the solute.^[^ ^31^ ^]^ Finally, it can be subjected to post‐processing treatments. This most often involves sintering, in cases where the slurry contains either ceramic or metallic particles, to densify and condense further the structure. Other postprocessing steps can include cross‐linking, when using natural polymers, or annealing.

Nelson et al.^[^ ^22^ ^]^ have proposed a simplified model for describing the kinetics of the templating process, as expressed for the critical freezing front velocity for an isolated particle. For velocities greater than *ν*_cr_, the particle will be entrapped in the ice front; whereas for velocities smaller than *v*_cr_, they will be rejected and become part of the ice lamellae. The variation of *v*_cr_ with process parameters is given by the following equation:^[^ ^22^ ^]^

(1) $$\nu_{\mathrm{cr}}=\frac{\Delta y_{0}d}{3\eta r}{\left(\frac{a_{0}}{d}\right)}^{n}$$

where Δ*y*_0_ is the free energy of the freezing system considering a single particle, *d* is the distance between the particle, and the freezing front, *η* is the dynamic viscosity of the liquid, *r* is the radius of the particle, *a*_0_ the distance between the molecules in the liquid, and *n* an empirical correction factor. All of these intrinsic factors will control and affect the freezing process, and therefore the microstructure of the resulting material.

**Figure 3:**
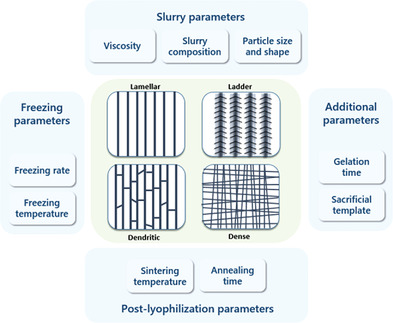
Factors affecting the resulting pore structure in ice templating processes. For optimizing the final material's properties, each of the parameters should be individually investigated.

The resulting pore structure will thus be defined by these intrinsic factors as well as external influences, as described in **Figure** **3**.

Ice templating differs from traditional freeze drying, in which the solution is casted into an uninsulated mold and placed in a freezer at a temperature below the solvents freezing point, to later be sublimated. This process yields an isotropic structure, with typically rounder pores, and has resulted in commercially available isotropic ice templated materials available in the market for the treatment of skin lesions, ulcers, and burns.^[^ ^32^ ^]^

Given the versatility of the technique, unidirectional ice templating allows the production of pore structures ranging from fully lamellar,^[^ ^33^ ^]^ to ladder‐like,^[^ ^34^ ^]^ dendritic,^[^ ^35^ ^]^ and dense microporosity.^[^ ^31^ ^]^ For example, for an alumina scaffold, using a ceramic powder with particle size 0.9 µm can produce an entirely columnar structure, whereas the use of platelet‐shaped particles with dimensions of 8 µm × 400 nm results in a dendritic morphology.^[^ ^36^ ^]^ Advantages and disadvantages of each morphology will depend on its intended application.

With so many factors at play affecting the templating kinetics, new solute or solvent systems to be investigated require a full study of the effects of the temperature and solid concentration in order to optimize the porous structure for the desired application.

**Figure 4:**
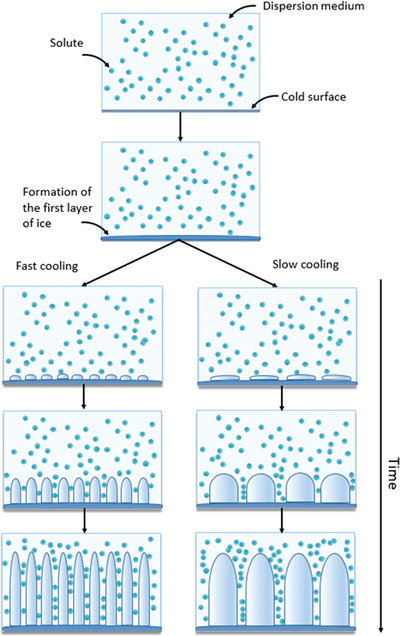
The effect of freezing rate on the formation of ice crystals during ice templating in a solute/solvent system. Increasing the freezing rate results in smaller ice crystals, which in turn yields a scaffold with smaller pores after lyophilization.^[^ ^22^ ^]^ The optimal pore size will depend on the intended application, hence the importance of selecting the appropriate freezing rate.

Pore size and structure play a key role in the biomechanical properties of tissue engineered scaffolds. They can affect cell attachment, orientation, tensile and compressive properties, permeability, and more. One of the more widely studied parameters for optimization of the scaffolds morphology is the freezing rate, as it is easy to modify and small changes can have significant effects. Increased cooling rates will result in smaller ice crystals and therefore smaller pores, as illustrated in **Figure** **4**.

Though no commercial devices exist for producing aligned ice templated biomaterials, experimental setups can range from simple Teflon molds put in contact with a surface cooled with liquid nitrogen and no control over either the freezing temperature or rate, to more advanced systems with temperature sensors and nitrogen flow control.^[^ ^37^ ^]^

**Figure 5:**
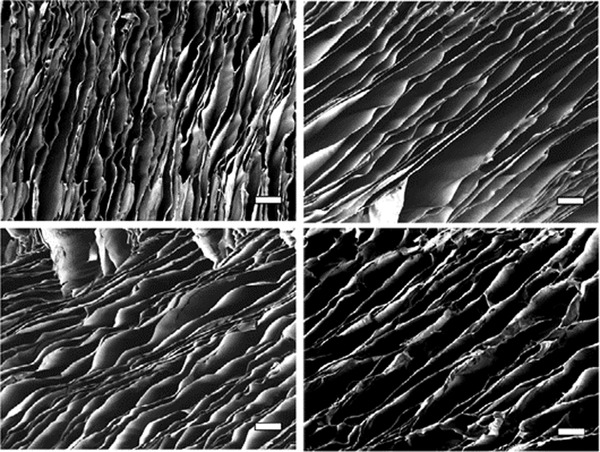
Reduction in pore size in an alginate ice templated scaffold with decreasing freezing temperature. Top left: −47 °C, top right: −33 °C, bottom left: −30 °C, bottom right: −25 °C. Freezing rate: 1 K mm^−1^. Scale bar: 200 µm. Reproduced under an open access Creative Common CC BY license.^[^ ^33^ ^]^ Copyright 2022, the Authors. Published by Wiley‐VCH GmbH.

Freezing temperature is an equally important parameter to optimize. One study^[^ ^33^ ^]^ developed a systematic investigation on the effects of precisely controlling the temperatures of both the top and bottom ends of an insulated mold while freezing a 2% w/w alginate solution. Maintaining a stable freezing gradient, but reducing the temperature of the bottom plate from −25 °C to −47 °C can have noticeable effects on the structure, decreasing the mean pore width from 144 to about 100 µm, as seen in **Figure** **5**.

Precise tailoring of the pore structure is key for the production of scaffolds with appropriate applications for different tissues in the musculoskeletal system. Understanding the role of each parameter at play in the ice templating process is the focus of much research being developed, in hopes to achieve superior biomaterials with high translational potential.

## 3 Bone

**Figure 6:**
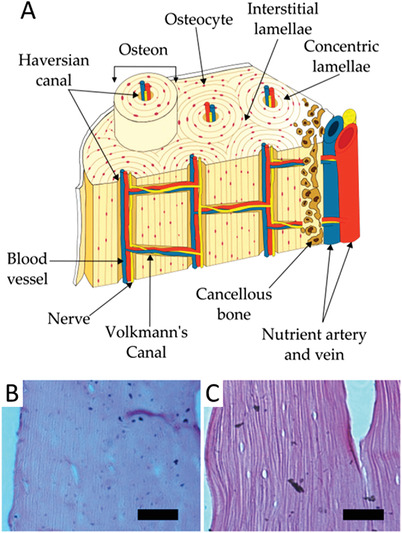
Microstructure of bone, highlighting the aligned structure of osteons and nutrient supply system to the osteocytes. A) Schematic of bone, detailing the different structures of trabecular and cortical bone. Reproduced under a Creative Commons CC‐BY license.^[^ ^45^ ^]^ Copyright 2019, the Authors. Published by MDPI. B) Hematoxylin and eosin staining of the femoral bone of a 2–4 week‐old rat. C) Hematoxylin and eosin staining of the femoral bone of an 8–10 week‐old rat, where the alignment of the extracellular matrix is visible. Reproduced under a Creative Commons CC‐BY license.^[^ ^46^ ^]^ Copyright 2019, the Authors. Published by MDPI.

Bone is the most widely researched tissue in the musculoskeletal system, and the second most transplanted tissue in the body, after blood.^[^ ^38^ ^]^ The two main structures present in bone are cancellous (also called trabecular) and cortical bone; they represent 20% and 80% of total bone tissue, respectively. Trabecular bone is predominantly located at the center of long bones, dispersed with the bone marrow, it has a porous and highly vascularized network structure. Cortical bone is dense and rigid, and functions as the main support for the body. It is composed of osteons, cylindrical structures of lamellae of bone tissue which surround the haversian canal, responsible for nutrient and fluid delivery.^[^ ^39^ ^]^ This structure is represented in **Figure** **6**. The composition of bone has been described as 60% HA, 10% water, and 30% proteins, which is mainly collagen type I.^[^ ^40^ ^]^

The compressive strength of bone has been found to depend on bone density,^[^ ^41^ ^]^ and therefore age;^[^ ^42^ ^]^ however ultimate compressive strength values for trabecular bone are in the range of 4−7 MPa and for cancellous bone in the range of 150−300 MPa.^[^ ^41^ ^]^ When designing the scaffold, pore size will be a key parameter, as it can affect cell attachment and proliferation. A study found higher cell numbers for scaffolds with a pore size of 325 µm, compared to scaffolds with pores in the range of 50−200 µm;^[^ ^43^ ^]^ although smaller pores in the range of 100−200 µm have presented improved osteocalcein levels compared to other pore sizes.^[^ ^44^ ^]^ Generally, a wide distribution of sizes could lead to a beneficial biological performance.

BTE strategies include allografts, autografts, xenografts, 3D printing, electrospinning and a variety of engineered scaffolds fabricated by foam replica and several other techniques. Autografts, the current gold standard treatment, presents several drawbacks: supply limitation, high failure rates in specific applications, donor side morbidity, and more. Metallic implants, widely used in long bone fractures and failures, are challenging due to their low degradation rates, and are usually not employed to fabricate scaffolds.

Anisotropic scaffolds are promising for BTE for a variety of reasons. On the one hand, the compressive properties of cortical bone are not isotropic: the compressive modulus of cortical bone has been found to be almost twice as high in the longitudinal direction as in the transverse direction.^[^ ^47^ ^]^ This can be replicated with aligned freeze casted structures, which can present directionally dependent and improved mechanical properties when compared to their anisotropic versions. On the other hand, these structures can be favorable to cells and nutrient infiltration,^[^ ^48^ ^]^ and can promote the “self‐seeding” of cells when in contact with a cell suspension. Based on the available literature we describe in the next sections the different material strategies relying on AIT.

### 3.1 Ceramics

Many different ceramic materials, including calcium phosphate ceramics, HA, and bioactive glasses, have been widely researched in the field of BTE, due to their excellent biocompatibility and osteoconductive properties. They can be processed in several different ways to achieve scaffolds with appropriate mechanical properties and morphology, including foam replica and additive manufacturing, among others. Ice templating provides a way of easily tuning the pore size, while maintaining an aligned ice structure suitable for cell attachment and vascularization.

The process involves the preparation and milling of a typically aqueous slurry, commonly accompanied by a dispersant and a binder, which subsequently undergoes unidirectional freezing. Following the lyophilization step, a sintering step is added to achieve a compact material. An overview of the research on aligned ice templated ceramic biomaterials can be found in **Table** **1**.

**Table 1:** Aligned, ice templated ceramic biomaterials for bone tissue engineering

Material	Investigated parameters	Porosity [%]	Mean pore size [µm]	Compressive strength [MPa]	Compressive modulus [Gpa]	Ref.
HA	Slurry concentration, cooling rate, sintering temperature	47–64	15–40	10–147	Not reported	[50]
HA	Solvent system composition	65–70	25–100	Not reported	Not reported	[51, 52]
HA	Particle size, cooling rate, sintering temperature	57–83	Not reported	1.7–15	Not reported	[75]
HA	Slurry concentration, cooling rate, sintering temperature	45–87	5–180	0.4–60	Not reported	[54]
HA–SiO_2_	SiO_2_ concentration	59–81	18–61	0.54–2.83	Not reported	[59]
FHA	Sintering temperature	39.4–42.1	17.9–23.9	5–13.5	0,1–0379	[57]
Biphasic calcium phosphate	Sintering temperature	43–45.9	21 (Sintering temperature: 1200 °C)	33.1–46.8	Not reported	[65]
CP	Freezing time	62–65	121–163	4.6–9.3	Not reported	[63]
HA, *β*‐TCP	Additive manufacturing combined with freeze casting	15	0.2–300	1.09	Not reported	[64]
*α*‐TCP	Treatments for phase conversion	59–88	9–23	1–5.29	Not reported	[66]
13‐93 BG	Freezing temperature, annealing time	20–60	6–120	16–180	4–25	[70]
13‐93 BG	Cooling rate	50	100	35–47	8–11	[71]
13‐93 BG	Solvent system, slurry concentration	55–62	25–100	5–50	0.4–1.2	[69]
66% SiO_2_, 5%P_2_O_5_, and 29%CaO BG	Slurry concentration	10–55	15–65	Not reported	Not reported	[73]
45S5 BG–carbon nanotubes	Carbon nanotubes concentration	63	20–100	2.08–4.56	0.115–0.266	[74]

#### 3.1.1 Hydroxyapatite

HA, as the main inorganic component in human bone, is naturally a good choice for BTE scaffolds, due to its excellent osteoconductivity and biocompatibility. However, HA scaffolds are still faced with challenges including achieving the necessary mechanical properties for load bearing applications and appropriate degradation rates.^[^ ^49^ ^]^

Ice templated HA scaffolds were first investigated by Deville et al.^[^ ^50^ ^]^ in a study that found that by optimizing the freezing rate, slurry concentration and sintering temperature, aligned freeze casted HA scaffold can be produced with compressive strengths up to 147 MPa, suitable for bearing loads. However, the porosity of 47% and pore size below 50 µm could difficult osteoblasts infiltration and bone growth.

The effect of pore size on cell viability and differentiation has been studied in HA scaffolds for pre‐osteoblastic cells.^[^ ^51^, ^52^ ^]^ Different pore sizes can be achieved by utilizing alternative solvent systems. Incorporating dioxane or glycerol, for example, can affect the nucleation kinetics of the ice crystals and yield completely different morphologies. Larger pore width, in the range of 100 µm, exhibit increased viability and differentiation potential when compared to pores of 25 µm. Furthermore, an increase of almost 2× could be found in total protein recovered from scaffolds with larger pores.

Particle size has also been proven to have an effect of HA scaffolds. Lower particle size can be correlated with increased porosity, reduced sintering‐induced shrinkage, and larger pore size; and is correlated with a lower compression strength.^[^ ^53^ ^]^

Sintering temperature has an important influence on the mechanical properties. Increasing the temperature from 1300 °C to 1350 °C can triple the compressive strength of a scaffold with particle size of 1.69 µm.^[^ ^54^ ^]^ Scanning electron microscopy (SEM) analysis has shown that the lower temperature results in incomplete sintering, with the pore walls exhibiting microporosity; compression testing reveals a decrease in mechanical properties.

Fluorohydroxyapatite (FHA), the result of a partial replacement of the hydroxyl groups in HA with fluoride, has been found to exhibit improved biocompatibility and thermal stability compared to HA.^[^ ^55^ ^]^ When tested in an in vivo rat model, FHA exhibits increased bone regeneration and decreased osteoclast activity compared to HA.^[^ ^56^ ^]^ Yin et al.^[^ ^57^ ^]^ studied the production of FHA freeze casted scaffolds from an aqueous slurry at three different sintering temperatures. The formation of *β*‐TCP (tricalcium phosphate) was found when sintering at 1450 °C, as well as a significant increase in compressive strength and cell viability compared to sintering at 1250 °C and 1350 °C. This indicates improved thermal stability of FHA compared to HA, which partially transforms into *α*‐TCP at just 1200 °C.

Incorporating SiO_2_ into the HA matrix has been found to have beneficial effects on the materials bioactivity.^[^ ^58^ ^]^ This is generally attributed to the incorporation of silanol groups, which improve the formation of apatite layers and favor the regeneration of bone. SiO_2_ addition influences the phase composition of the material, with increasing amount of SiO_2_ leading to the formation of *α*‐TCP and *β*‐TCP phases. The gradual addition of SiO_2_ to a 20% HA slurry up to 5% results in an increase in the porosity and pore size of an ice templated scaffold, whereas a 10% content reduces drastically both parameters.^[^ ^59^ ^]^ This could be explained due to the complete transformation of HA into TCP and condensation of the structure after sintering. After immersion in simulated body fluid (SBF), the 5% SiO_2_ exhibited the thickest bone‐like layer of apatite on its surface, whereas the 10% exhibited high dissolution rates and a granular apatite layer. One study^[^ ^60^ ^]^ found that adding 2.5% of SiO_2_ particles into different concentrations of HA slurries had no marked effect on both porosity and pore size; however, a significant effect on compressive strength could be found for a HA concentration of 35%, going from 17.6 MPa without silica to 29.3 MPa after the addition of SiO_2_.

The reviewed HA scaffolds still present limitations, as their low biodegradability remains a challenge.^[^ ^61^ ^]^ Though much work has been done into investigating the influence of the process parameters in AIT into the properties of the final scaffold, more research is needed into the biological performance of these scaffolds, as a HA aligned porous scaffold is yet to be tested in an in vivo model. Achieving the appropriate mechanical properties for applications in cortical bone while maintaining suitable porosity also remains a challenge.

#### 3.1.2 Calcium Phosphates

Calcium phosphate is a popular material for BTE, mainly due to its chemical similarity with HA, as well as biocompatibility and osteoinductive properties.^[^ ^62^ ^]^ A CaP freeze casted scaffold was first produced by Soon et al.,^[^ ^63^ ^]^ using camphene as part of the solvent system, looking to produce a material with enhanced mechanical properties when compared to isotropic structures. Camphene is frequently used for freeze casting ceramic materials, due to its much more moderate freezing temperature, with a melting point of 51.5 °C, and its capacity to from crystals which overgrow after freezing for prolonged amounts of time, thus forming larger pores after drying.^[^ ^36^ ^]^ By modifying the freezing time, different pore structures can be achieved, ranging from 121 µm after 1 day freezing to 163 µm after 3 days. In the first stages of freezing, camphene dendrites are found to grow in the direction of freezing, and later found to enlarge in the perpendicular direction, increasing pore width. Compressive strength values decrease with increasing pore size, from 9.3 to 6.2 MPa.

Additive manufacturing and freeze casting have been combined in recent years hoping to obtain aligned porous materials with complex geometries.^[^ ^1^ ^]^ This approach was used in order to obtain both HA and TCP multihierarchical scaffolds, with macropores of 0.3 mm, produced by the 3D printed template, micropores of 4 µm resulting from the freeze casting process, and 200 nm nanopores.^[^ ^64^ ^]^ This multileveled structure allows pre‐osteoblastic MC3T3‐E1 cells to penetrate up to 2.76 mm into the scaffold, compared to just 0.36 mm for traditional aligned freeze casted scaffolds. The resulting materials possess appropriate mechanical properties for applications in cancellous bone, and porosities between 65% and 75%.

Biphasic calcium phosphate has emerged as an interesting material for BTE. A combination of HA and *β*‐TCP, it takes advantage of HA's excellent biocompatibility and *β*‐TCP's faster degradation kinetics and bone absorption rates. Using a complex solvent system, including tert‐butyl alcohol and citric acid, completely lamellar structures can be produced using biphasic calcium phosphate, compared to the dendritic structures formed by simpler systems of water or camphene.^[^ ^65^ ^]^ Scaffolds were produced with pore size around 20 µm, at two different sintering temperatures: 1200 °C and 1300 °C. Negligible differences were found in the pore size and porosities of the two versions; however, a significant increase can be seen in the compressive strength for the 1300 °C sintered scaffold, which could be attributed to a densification process occurring at this temperature. Interestingly, formation of *α*‐TCP could be detected in the 1300 °C sintered scaffold, that was quickly released in the first week, leading to very similar mechanical behavior in both scaffolds after the second week.

**Figure 7:**
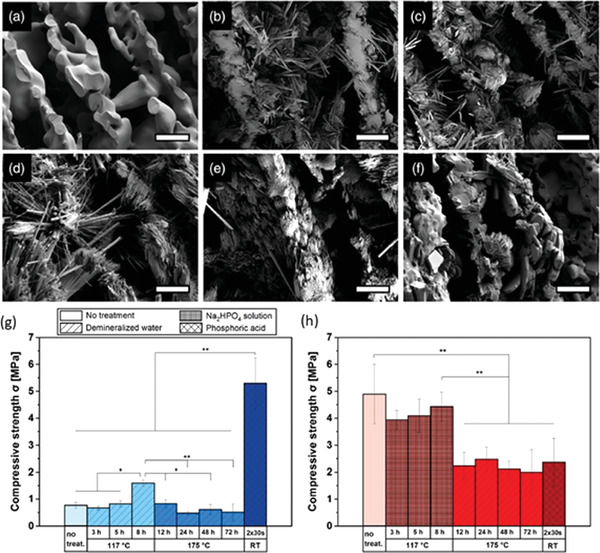
Effect of different post‐sintering treatment on the morphology and mechanical properties of an aligned *α*‐ tricalcium phosphate (TCP) scaffold. a) Lamellar structure of sintered sample, untreated. b,c) Calcium‐deficient hydroxyapatite (CDHA) crystal formation after hydrothermal treatment for 3 and 8 h. d,e) CDHA crystal bundles formed after incubation at 175 °C for 12 and 72 h, respectively. f) Brushite crystal formation after immersion in acid solution. Scale bar: 15 µm. g,h) Compressive strengths of unsintered and sintered samples, respectively. ^*^ *p* < 0.05, ^**^ *p* < 0.001). Reproduced under an open access Creative Common CC BY license.^[^ ^66^ ^]^ Copyright 2021, the Authors. Published by Wiley‐VCH GmbH.

Other calcium phosphate phases, such as monetite, brushite, and calcium‐deficient hydroxyapatite (CDHA), could be interesting for BTE due to their improved absorption rates and degradation kinetics compared to HA and TCP. However, they can present low stability at high temperatures, impeding their sintering and thus their processability. In order to circumvent this issue, a study proposed strategies involving producing a freeze casted *α*‐TCP scaffold, sintering, and either immersing into a phosphoric acid solution (to produce monetite/brushite) for 3–72 h, or hydrothermal treatment 117 °C up to 8 h, and 175 °C up to 72 h (to produce CDHA).^[^ ^66^ ^]^ Complete conversion into CDHA could be achieved with hydrothermal treatment, and partial conversion was found via the acid treatment, in nonsintered samples into monetite, and in sintered samples into brushite. Immersion into phosphoric acid was found to decrease the scaffolds porosity, from 85% to 79% in the nonsintered scaffolds and 75% to 59% in their sintered versions. The effects of the phase conversions can be clearly appreciated via SEM images, where the CDHA appear with a needle like morphology, the monetite forms around the lamellae in platelet‐shaped crystals, and the brushite was formed into bundles of crystals on the sides of the pores (**Figure** **7**). For nonsintered samples, the acid treatment has the largest influence on the materials compressive properties, going from 1 to 5.29 MPa, a value comparable to sintered samples. The biocompatibility and cellular response to these biomaterials remain to be studied.

#### 3.1.3 Bioactive Glass

Bioactive glass, a material first developed by Larry Hench in 1969, is a highly versatile and interesting platform for BTE scaffolds.^[^ ^67^ ^]^ By forming a calcium and silicon network, these glasses exhibit not only excellent biocompatibility, but also have the capacity to form HA in vitro and in vivo, making them excellent candidates for applications in BTE.^[^ ^68^ ^]^ This versatile material yields itself to the production of aligned ice templated scaffolds through a slurry, which can be aqueous or contain different solvents. The incorporation of such solvents, such as dioxane, has been investigated to affect the pore structure of scaffolds produced from 13 to 93 glass, with a composition in weight percentage of 53% SiO_2_, 6% Na_2_O, 12% K_2_O, 5% MgO, 20% CaO, 4% P_2_O_5_.^[^ ^52^, ^69^ ^]^ Pores in the range of 100 µm present significantly higher rates of cell proliferation and ALP activity compared to pores of 25 µm.

Camphene was similarly studied, in order to investigate the possibility of achieving a wider range of pore sizes. Liu et al.^[^ ^70^ ^]^ studied the effects of incorporating camphene and adding an annealing step to the scaffold fabrication process. They demonstrated that dispersant concentration can drastically alter the solutions viscosity, which will in turn affect the final pore structure. They further find that adding an annealing step can significantly affect the scaffolds pore size, going from 40 µm without annealing to 160 µm after annealing for 72 h. After a sintering step, however, the pores are again reduced to a mean size of 120 µm. The compressive strength of the material decreases with increasing annealing time, indicating an inverse relation between pore size and compressive properties.

In a further study,^[^ ^71^ ^]^ they analyze the effect of maintaining a stable cooling rate during freezing on the materials properties. As predicted, the slowest cooling rate leads to larger pores, both when compared to faster rates and maintaining a fixed freezing temperature on the cool substrate (and not controlling the freezing rate). Furthermore, cooling at a faster rate leads to a more homogeneous scaffold, with similar pore sizes all throughout the freezing direction; whereas the fixed temperature scaffold presents up to three times larger pores on the top than at the bottom. The freezing rate of 7 °C min^−1^ was chosen as optimal, and the scaffolds were further tested in vivo on a rat model, compared to an isotropic scaffold produced via foam replication technique, with a higher porosity and lower compressive properties (similar to trabecular bone).^[^ ^72^ ^]^ A defect was created in the parietal bone, and animals were sacrificed after 12 and 24 weeks. After 12 weeks, the mineralized area of new bone is around 60% for the aligned and 30% for the isotropic scaffolds. The improvement in bone formation for the oriented scaffolds could be an indication that the aligned morphology of the pores is better for osteoconductivity than the random morphology of the foam replica scaffolds.

A scaffold produced from 66% SiO_2_, 5% P_2_O_5_, and 29% CaO glass was found to exhibit a linear decrease in porosity and pore size with increasing powder concentration, with similar trends found in many studies for ceramic freeze casted materials.^[^ ^73^ ^]^ Strong bioactivity was detected by measurement of ALP activity, indicating potential for stimulating bone cell differentiation; and apatite formation was verified by Fourier transform infrared spectroscopy (FTIR) and atomic emission spectroscopy analysis of SBF after immersion.

Carbon nanotubes are an alternative to reinforcing the mechanical properties of a BG scaffold for applications in load bearing sites.^[^ ^74^ ^]^ By modulating the total concentration of BG in the material, both its microstructure and mechanical properties can be tuned to values appropriate for BTE applications. A concentration of 0.25% of multiwalled carbon nanotubes can improve the compressive stress of a 45S5 bioactive glass freeze casted scaffold from 2.09 to 4.56 MPa, while more than doubling its elastic modulus, presenting a viable alternative for applications in trabecular bone.

Bioactive glass scaffolds have exhibited promising results for applications in BTE, as they are the only ceramic material that has been successfully tested in an in vivo model, where significant improvements in tissue regeneration were found compared to isotropic scaffolds produced by an alternative technique.^[^ ^72^ ^]^ Though their suitability in non‐load bearing applications has been demonstrated, further work should focus on improving their mechanical properties to match those of cortical bone.

### 3.2 Polymer and Composites

Polymeric biomaterials have been widely studied for a variety of applications and tissues. Thanks to their versatility and large array of tunable properties they are a very attractive alternative for ice templated biomaterials. Blends of both natural and synthetic polymers, including polycaprolactone (PCL)/zein^[^ ^76^ ^]^ and poly(lactic‐co‐glycolic acid) (PLGA)/gelatin^[^ ^77^ ^]^ have both been proposed for the development of tissue engineered scaffolds. These seek to take advantage of the biocompatibility and hydrophilicity of the natural polymer, as well as the mechanical properties and tunable degradation kinetics of the synthetic.

An ice templated scaffold was produced from PCL and zein, with a solvent blend of chloroform, ethanol, and acetic acid, in order to study the drug release kinetics of tetracycline hydrochloride (TCH).^[^ ^76^ ^]^ Using PCL alone, 90% of the drug was released in 24 h, whereas the addition of zein resulted in 66% of the total amount of TCH released in the same time. FTIR analysis reveals shifts in the TCH‐zein spectra, indicating interactions between the protein and the drug, which could explain its delayed release. The total amount of TCH in the PCL‐zein scaffold is released after 14 days, indicating suitability for applications requiring sustained drug release.

However, though blends such as these present many interesting properties, a purely polymeric freeze casted material has yet to achieve the appropriate mechanical properties required for applications in BTE. This has led to the use of a secondary material, such as HA, bioactive glass, and others, to reinforce and functionalize the scaffolds. An overview of the investigated composites for AIT can be found in **Table** **2**.

**Table 2:** Aligned, ice templated composite biomaterials for bone tissue engineering

Polymer	Additives	Investigated parameters	Porosity [%]	Pore width [µm]	Compressive strength [MPa]	Compressive modulus [MPa]	Ref.
Gelatin	Ti6Al4V	Freezing temperature	49.9–75.2	10–430	5–400	610–2300	[79]
Gelatin	BGNPs	BGNPs concentration	98	40–60	1.48–1.69	Not reported	[91]
Gelatin	HA, DXM loaded PLGA microspheres	HA concentration, freezing rate	95	20–60	1.7–2.5	Not reported	[78]
Gelatin, collagen	HA	Collagen, HA concentration	27.08–36.99	10.61–61.41	0.01–0.07	0.04–0.09	[92]
Chitosan	BGNPs	BGNPs concentration	88.98–96.6	Not reported	0.034–0.419	0.41–10.77	[83]
Chitosan	GO	GO concentration	Not reported	7.5–40	0.068–0.14	0.4–1.6	[82]
Chitosan	HA	HA addition, freezing direction	90.23–97.63	84–98	0.1–0.6	Not reported	[84]
Chitosan, gelatin	45S5 Bioactive glass	Freezing rate, gelation time	74.36–76.82	73–120	2.2–3.1	19.4–46.0	[93]
Collagen	Apatite	Compression time, freezing temperature	94	63.8–344	12	36.4	[86]
Silk	Silica	Cooling rate, cooling temperature	91–94	0.52–17.84	0.39–1.6	4.03–7.3	[94]
Silk, PEG	HA	HA concentration	Not reported	Not reported	0.03–0.09	0.081–0.191	[89]
Starch	Cellulose nanofibers (CNFs), HA	HA, CNFs concentration	95	80–292	Not reported	0.845–1.768	[95]
PCL	58S BG	Slurry composition	59.2–79.0	45–120	0.15–4.1	Not reported	[87]
PCL	BGNPs, SIM	Plasma treatment, SIM immobilization	96	Not reported	2–4	Not reported	[9]
PCL	*β*‐TCP	Cooling rate, slurry concentration	50.1–85.6	8–20	0.5–40	Not reported	[96]
PLGA	CPC	Slurry composition	58.5	100−200	5	Not reported	[90]
PLGA	CPC, Collagen	Slurry composition	58.5–63.8	100–150	5	Not reported	[97]
PLGA	CPC, PRP	Slurry composition	Not reported	100–150	Not reported	Not reported	[88]

#### 3.2.1 Natural Polymer Composites

##### Gelatin

Frequently chosen due to its biocompatibility, degradability, and low cost, it is often reinforced and functionalized with a secondary phase in order to improve its osteogenic potential and mechanical properties. The incorporation of HA, titanium, and bioactive glass have all been studied.

A gelatin‐HA aligned composite scaffold was successfully functionalized with the addition of dexamethasone‐loaded PLGA microspheres,^[^ ^78^ ^]^ and the effects of HA concentration and freezing rates were evaluated. A decrease in pore size resulted from both an increasing powder concentration and freezing rate, with smaller pore sizes leading to slower hydrolytic degradation. Drug release studies show that both cooling rate and HA concentration play a big role in release kinetics, where the scaffolds with the highest HA concentration and fastest cooling release dexamethasone significantly slower than the other versions. This phenomenon can be linked to the smaller pore size and slower degradation behavior.

The incorporation of gelatin has been studied to help the dispersion of particles and prevent sedimentation, resulting in a homogeneous scaffold. A Ti6Al4V‐gelatin composite was found to present compressive properties in the range of cortical bone, suitable for load bearing applications.^[^ ^79^ ^]^ The influence of gelatin on the pore structure was evident in SEM images, with pores acquiring a honeycomb‐like morphology and increased interconnectivity when compared to using other binders. By applying a freezing temperature of −15 °C, an average porosity of over 70% could be achieved, and an elastic modulus similar to that of cortical bone was measured, which would prevent stress shielding effects. Though highly promising, further biocompatibility and osteointegration studies would be necessary for a better understanding of the biological performance of the scaffold.

##### Chitosan

Chitosan, a linear polysaccharide derived from chitin, has many interesting properties which make it an attractive option for application in tissue engineering. Among them are its biocompatibility, protein adsorption, and mild antibacterial effect.^[^ ^80^ ^]^ Chitosan has been combined with bioactive glass, HA, and graphene oxide (GO), in hopes of improving its bioactivity and mechanical properties.

The incorporation of GO to a chitosan matrix can improve the stability of the material, by forming electrostatic interactions and bonds between the functional groups of GO and the polymer.^[^ ^81^ ^]^ Concentrations between 0.5% and 2% of GO, added to a chitosan solution, can produce unidirectional scaffolds with pore sizes between 7.5 and 40 µm.^[^ ^82^ ^]^ The resulting material exhibits significantly increased compressive properties and shape memory. MC3T3‐E1 cells seeded in aligned scaffolds show alignment in the direction of the grooves, with no alignment detected for random scaffolds.

Incorporating bioactive glass nanoparticles (BGNPs) into a chitosan scaffold, with concentrations between 10% and 50% (with respect to the chitosan concentration) results in an improvement in the mechanical properties of the material.^[^ ^83^ ^]^ Furthermore, apatite formation can be verified through SEM images, with a 30% content resulting in the highest amount of total apatite formation, possibly due to a higher absorption capacity.

A chitosan‐HA composite with radially oriented pores, rather than vertical, has been proposed as an alternative freeze casting method, in hopes to improve migration of osteocytes and stem cells from surrounding tissue, as well as inhibit fibroblast recruitment from the injured site.^[^ ^84^ ^]^ These centrosymmetric structures have also been found to exhibit improvements in their strains at break in titanium biomaterials when compared to uniaxially aligned materials, though ultimate compressive strength and Young's modulus remain lower. Seeding the scaffolds with rat bone marrow mesenchymal stem cells (MSCs) revealed no differences in cell proliferation or ALP activity values between the vertical and radial scaffolds, as well as no cytotoxic effect from the addition of HA to the chitosan matrix. An in vivo rat subcutaneous implantation model as well as a rabbit femoral defect model were selected to determine any differences in performance between radial and aligned scaffolds. Both structures were conductive to the formation of fibrotic tissue into the pore channels, exhibiting foreign body reactions in the subcutaneous implantation. However, significantly more new bone volume was found for the radial scaffolds than for their aligned versions in the femoral defect model, as well as an inhibition of fibrous tissue from penetrating deep into the material, indicating that centrosymmetric biomaterials could be promising for applications in BTE.

##### Other Natural Polymers

Collagen, silk, and corn starch have also been researched for potential applications as composite materials in freeze casted scaffolds for BTE.

A variation on the aligned freeze casting technique was presented where an array of rods were introduced into the mold, arranged in perpendicular direction to that of freezing, in order to improve the interconnectivity between the pores.^[^ ^85^ ^]^ The effect of incorporating horizontal channels into the structure on the mechanical properties was not investigated. Using these custom molds, a silk aligned scaffold was produced and further coated with apatite by immersion in 10× SBF, to improve the materials bioactivity, followed by loading with bone morphogenetic protein 2 (BMP‐2) solution. Scaffolds were tested on a rat femur critical defect model, where the animals were sacrificed 10 weeks following the operation. Coated and loaded scaffolds exhibited a significant increase in newly formed bone tissue when compared to the unloaded and uncoated scaffolds, showing that the BMP‐2 and the apatite coating exhibit a synergistic effect for bone formation.

The self‐compression of a hydrogel, the process in which a hydrogel remains at room temperature for a certain amount of time releasing water due to its own weight, can have an effect of the resulting pore structure of an ice templated material.^[^ ^86^ ^]^ An aligned macroporous scaffold from a collagen‐apatite hydrogel was allowed to undergo self‐compression for 5, 20, and 45 min before the freezing process, where three different freezing temperatures were evaluated. All scaffolds were cross‐linked with *N*‐(3‐dimethylaminopropyl)‐*N*’‐ethylcarbodiimide (EDC) and freeze dried a second time before testing. X‐Ray Diffraction (XRD) analysis confirmed the deposition of apatite on the scaffold. A stark decrease in pore size can be found for increasing self‐compression times, where 5 min of compression yields a mean pore size of 344 µm, whereas for 20 min it is 173 µm. This demonstrates another possible alternative for easily modifying the scaffolds final pore structure. The effect of the freezing temperature on the pore size was similarly evaluated, with mean values of 173, 146, and 63.8 for −25 °C, −80 °C and −196 °C, respectively. The influence of the different pore sizes on cell proliferation was not studied.

#### 3.2.2 Synthetic Polymer Composites

Multiple synthetic polymers have been studied for potential applications in BTE with an ice templated approach, including polyethylene glycol (PEG), PCL, and PLGA, due to their many attractive properties, including tunable degradation kinetics and lack of batch variability.^[^ ^87^, ^88^, ^89^ ^]^ For applications in freeze casted scaffolds, they are often combined with ceramic particles, in hopes to complement the bioactivity of ceramics with the polymers mechanical properties and biocompatibility.

**Figure 8:**
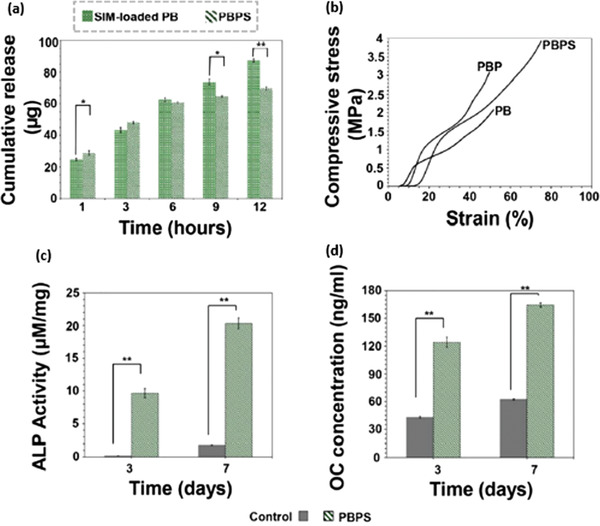
Investigation of oxygen plasma treatment for simvastatin (SIM) immobilization on a polycaprolactone (PCL) scaffold. a) Cumulative release of SIM from the scaffolds. b) Stress–strain curve for the different conditions. c) ALP activity of the bare and SIM loaded scaffolds. d) Expression of osteogenic biomarker of the bare and SIM loaded scaffolds. ^*^ *p* < 0.05, ^**^ *p* < 0.005). Reproduced with permission.^[^ ^9^ ^]^ Copyright 2021 Elsevier.

A PCL‐BGNPs aligned scaffold was treated with oxygen plasma for the immobilization of simvastatin (SIM), a drug capable of stimulating the excretion of BMP‐2, on its surface (**Figure** **8**).^[^ ^9^ ^]^ Treating the surface is beneficial not only for drug delivery purposes, but also for improving the hydrophilicity of the material, which is directly related to its ability to adhere cells on its surface. By incorporating carboxyl and hydroxyl groups, the liquid absorption capacity of the scaffold is also greatly improved. The plasma treatment was found to increase the mechanical properties up to 1.5 times compared to the untreated scaffold, producing a material within the appropriate range for applications in cancellous bone. Drug release was analyzed by comparing the plasma treated, drug loaded scaffold, with an untreated scaffold soaked in SIM solution. The treated scaffold results in less than 70% of total drug released after 12 h, whereas the soaked material reaches 90%, indicating that oxygen plasma treatment can allow for a more sustained drug release. Furthermore, the osteogenic capacity of SIM was verified through ALP activity analysis, demonstrating a fourfold increase compared to the control cells. Osteocalcin marker levels were evaluated as a marker of osteogenesis, revealing a significant increase in values after 3 and 7 days, compared to the control.

A calcium phosphate cement (CPC) PLGA (CPC‐PLGA) composite was developed by He et al.,^[^ ^90^ ^]^ seeking to synergistically combine the osteoconductive and bioactive properties of CPC with the mechanical properties and biodegradation rate of PLGA. This promising composite material was found to be highly biocompatible and possess comparable mechanical properties to trabecular bone, though the PLGA component was found to inhibit osteoconductivity. By introducing a platelet rich plasma (PRP) coating,^[^ ^88^ ^]^ the release of different growth factors was stimulated, including BMP‐2 and VEGF, associated with bone healing. Rat MSCs were found to proliferate at two times the rate of the control scaffold after 7 days with the application of PRP, and an increase of ALP activity was detected after 14 days. The PRP coated biomaterial was tested on two different in vivo rabbit models, in the first case by creating a critical size defect in the femora, in the second by generating a 15 mm defect in the radius. Postsurgical analysis detected the formation of mineralized bone in both the control (CPC‐PLGA) and test groups (CPC‐PLGA‐PRP) in the femoral defects, however significantly more vascular growth was found for the PRP group. In the case of the radial defects, the differences were more marked between the experimental groups. The PRP loaded scaffolds showed increased capacity of generating new bone tissue, with evidence of adherence of the material to the host tissue in as little as 6 weeks.

### 3.3 Metals

Solid, nonporous metallic implants are commonly used for load bearing bone applications. However, they present some disadvantages, including generating stress shielding, which is the loss of native bone density due to the presence of a much stronger foreign material.^[^ ^98^ ^]^ This can be alleviated with the incorporation of pores into the implant, to reduce its mechanical properties and more closely match them to those of natural bone. By aligning the pores in one direction as opposed to randomly, the mechanical properties and the osteoconductivity can be improved. The development of ice templated metallic materials has been widely studied from thermodynamic and structural points of view, including applications for biomaterials and tissue engineering. Systematic investigations have been conducted on the influence of solid content, freezing temperature, and slurry composition for different metallic components.^[^ ^99^ ^]^

TiH_2_ can release hydrogen and inhibit the formation of titanium oxide, making it an interesting option for applications in freeze casting. By varying the TiH_2_ content between 20% and 30% in an aqueous slurry, and the freezing temperature between −10 °C and −50 °C, progressive changes in the pore structure were found.^[^ ^100^ ^]^ The pore walls increase their thickness and pore width decreases with increasing solid concentration, whereas porosity is found to decrease significantly both with lower temperatures and higher solid content, ranging from 73% for a 20% TiH_2_ scaffold frozen at −10 °C, to 15% for a 30% scaffold frozen at −50 °C. When studying the mechanical properties, a correlation is found between porosity and maximum compressive stress. For samples with 48% porosity, the ultimate compression strength reaches 268 MPa at a strain of 5%, which could indicate its suitability for load bearing tissue. The mechanical properties decrease quickly with an increase in porosity, with a value of 20 MPa for a material with 73% porosity.

Li et al.^[^ ^101^ ^]^ have investigated the fabrication of a Ti_6_Al_4_V scaffold produced from a SiC fiber‐containing slurry, in hopes of obtaining a scaffold with high porosity and optimal compressive properties. SiC fibers are a common reinforcing agent due to their excellent mechanical properties and good biocompatibility, and when used in ice templating, they could form bridges between the lamellar walls to improve the material properties. Slurries were prepared with different proportions of metal to fibers, and unidirectionally frozen with a metal plate at −10 °C. After sintering at high temperature, a reaction occurs between the Ti and the SiC fibers, forming TiC, which can be verified via XRD analysis. The formation of interlamellar bridges can be confirmed via SEM images. The SiC has a strong influence on the porosity, increasing from 63% for pure Ti6Al4V scaffolds, to 73% for scaffolds with 10% SiC. When analyzing the compressive properties of materials with different SiC content, it's found that moderate amounts of fibers, in the range of 2–5%, improve the overall mechanical behavior of the materials, allowing partial buckling and cracks to appear after 4% strain. For materials with 10% SiC content, it was found that the rigidity of the material increases to the point where it breaks at strains lower than 2%. The material containing 2% fibers possesses the highest strength‐to‐modulus ratio of all the tested variants, which signifies a very promising alternative for reducing stress shielding effects.

Ti_6_Al_4_V alloy was also freeze casted with the aid of a WS_2_ template, which was removed following the lyophilization step.^[^ ^102^ ^]^ Different pore sizes were achieved by modifying the composition of the slurry. Pores sizes ranging from 84 to 217 um exhibit distinct mechanical behavior, with pores of 145 um resulting in the highest compressive strength and modulus. Values of 142 and 5 Gpa were achieved for compressive strength and compressive modulus respectively, indicating that the freeze cast biomaterial could be suitable for load bearing applications.

Overall, it has been demonstrated that metallic scaffolds with appropriate properties for cancellous bone can be obtained through ice templating. However, very little is mentioned about the hydrophilicity and cell attachment performance of these materials. The surface of metallic implants often requires treatments to improve osteoconductivity and bone formation,^[^ ^103^ ^]^ further work is required to fully understand the potential of metallic ice templated scaffold in a biological environment.

## 4 Tendon

Tendon is a hypocellular and hypovascular tissue, capable of resisting high amounts of tension and transmitting forces from muscles to bone. Tendons are subjected to very high levels of stress, and due to a combination of trauma, overuse, and aging, tendon injuries are extremely common.^[^ ^104^ ^]^ Rotator cuff tears alone affect over 50% of people over the age of 60 and it has been estimated that anywhere between 20% and 94% of surgical repairs fail, usually within the first 6 months.^[^ ^16^ ^]^ This highlights a need for improved tissue engineering strategies to solve this impending need.

**Figure 9:**
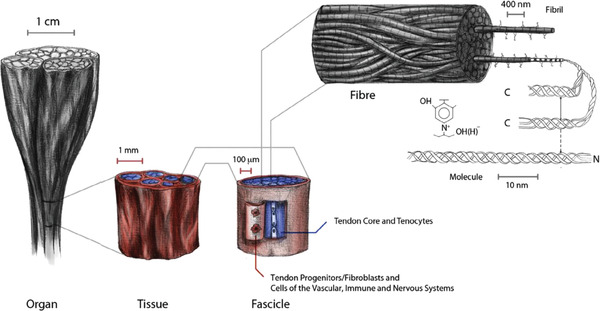
Tendon microstructure and individual units that make up the tendon extracellular matrix. Collagen type I is arranged into nanometric fibrils, which in turn are organized into fibers. These fibers are key to the mechanical properties of tendon tissue. They are arranged into fascicles, which are surrounded by a fascicular membrane containing a diverse cell population. Reproduced with permission.^[^ ^108^ ^]^ Copyright 2017, Elsevier.

The microstructure of a tendon, though completely different to that of bone, could also be replicated with the use of aligned freeze dried biomaterials. In tendons, collagen type I strands are organized into fibrils, which in turn arrange themselves into fibers, that comprise the structural unit of a tendon fascicle, where blood vessels and nerves are present. Fascicles organize themselves into the larger tendon structure, separated by an interfascicular membrane. This structure plays a key role in the biomechanical behavior of tendons, giving it a very high tensile strength, while also allowing for optimal cell adhesion and proliferation.^[^ ^105^ ^]^ The structure is represented in **Figure** **9**. The mechanical properties of tendon will vary widely depending on the specific tendon, the section of tendon that is measured, health, and age of the measured tendon. For supraspinatus tendon, values have been reported in the range of 2.8−150 MPa for ultimate tensile stress;^[^ ^106^ ^]^ the values are in the range of 20–100 MPa for Achilles tendon.^[^ ^107^ ^]^

This highly hierarchical structure yields itself to be mimicked utilizing these biomaterials. An aligned topography is vital for material applications in tendon, given that tenocytes are known to respond to aligned topographies by increasing their proliferation and collagen production.^[^ ^27^ ^]^ Immune response could also be improved, when compared to isotropic scaffolds, since anisotropic structures can induce the differentiation of macrophages into their M2 phenotype, producing an anti‐inflammatory response.

For the fabrication of tendon scaffolds, the usual material of choice has been collagen, given it is the principal component of tendon extracellular matrix and possesses good biocompatibility and biodegradability. Silk has also attracted much interest,^[^ ^109^ ^]^ due to its excellent mechanical properties and significantly lower cost.^[^ ^110^ ^]^ The use of these natural polymers, though presenting many advantages, signifies a big challenge in mimicking the tendons native properties. For this reason, much work has focused on tuning the processing parameters of the technique in order to maximize the materials tensile properties. It is important to note, however, that even many commercially available devices for tendon augmentation fail to match the right mechanical properties, indicating that further work is required in this field.^[^ ^111^ ^]^

Increasing the total polymer concentration of a collagen‐glycosaminoglycan (GAG) scaffold, and therefore the relative density of the material (calculated by dividing the scaffolds density by the theoretical density of collagen), has been linked to a significant increase of the tensile properties.^[^ ^112^ ^]^ However, the range of ultimate tensile strength values reported was 20–200 kPa, at least one order of magnitude away from the target values. The denser scaffolds also exhibit increased cell viability and cell number, as well as improved metabolic activity and collagen production. When looking at the effect of freezing temperature on tenocyte performance, no significant differences could be detected for cell proliferation on collagen‐GAG scaffolds frozen at −10 °C, −40 °C, and −60 °C, with corresponding pore sizes of 243, 152, and 55 µm;^[^ ^27^ ^]^ however, utilizing the higher temperature, and therefore achieving the largest pore size, allows for cell migration deeper into the scaffold and a more even cell dispersion throughout. These benefits could be counteracted with a decrease in mechanical properties (values not reported) and inhibited cell adhesion. Though these scaffolds lack the appropriate mechanical properties for applications in tendon, they have provided important insight into the influence of pore size on tenocyte viability.

The effect of coating collagen scaffolds with a fibrin gel was also studied.^[^ ^113^ ^]^ It is known that fibrin can facilitate the alignment of tendon cells, however fibrin alone as a gel is not stable enough to support tendon repair.^[^ ^114^ ^]^ By combining the two structures, a synergistic effect was found, where cell number improved significantly, yielding a more evenly dispersed cell density throughout the scaffold, and the expression of tenogenic markers such as tenomodulin increased. Interestingly, the addition of fibrin gel resulted in a lower expression of fibronectin, a key protein in cell growth and migration. This could be attributed to the fact that the fibrin gel provides the necessary topography for cells to migrate through the material, without the need for synthesis of fibronectin.

**Figure 10:**
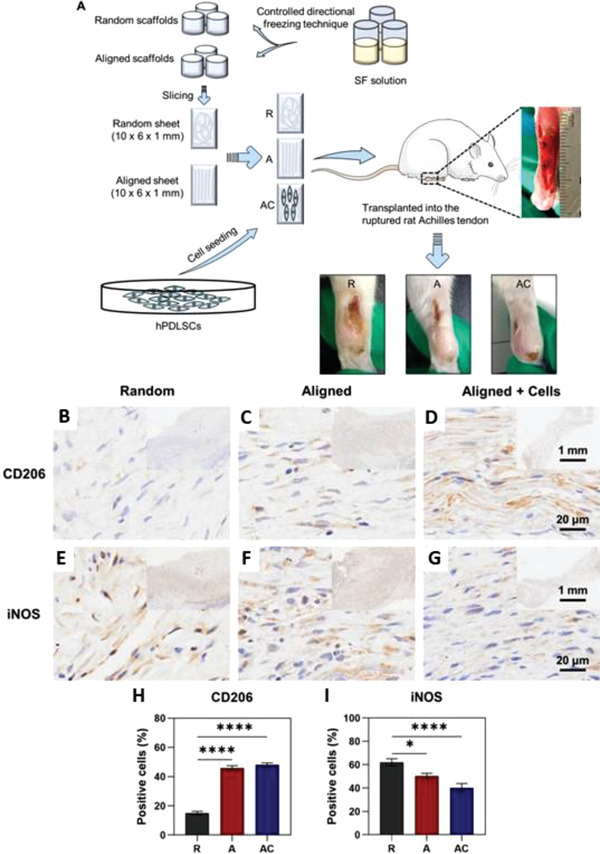
Silk fibroin aligned scaffold tested on an in vivo tendon rat model. a) Schematic representation of the experimental steps. b–d) Immunohistochemical staining with C206 antibody (anti‐inflammatory marker) for all three experimental conditions. e–g) Immunohistochemical staining with iNOS antibody (pro‐inflammatory marker) for all three experimental conditions. h) Percentage of C206 positive cells. i) Percentage of iNOS positive cells. ^*^ *p* < 0.05, ^****^ *p* < 0.0001. Reproduced under an open access Creative Common CC BY license.^[^ ^117^ ^]^ Copyright 2021, the Authors. Published by BioMed Central.

Macrophage polarization plays a key role in tendon healing. In the early stages of repair, M1 macrophages are recruited to the injured site by resident immune cells, which then promote a pro‐inflammatory response. After about 2 weeks, M2 macrophages start to predominate and favor an anti‐inflammatory behavior, reducing the presence of scar tissue.^[^ ^29^ ^]^ At the same time, fibroblasts and other cells synthetize collagen for the repair of the ECM. An excess of M1 phenotype expression could lead to chronic inflammation;^[^ ^115^ ^]^ whereas an excess of M2 expression can be linked to fibrosis.^[^ ^116^ ^]^ In an effort to study the effect of surface morphology on the immune response, silk fibroin scaffolds produced via both random and AIT methods were seeded with human periodontal ligament stem cells and subsequently implanted into a rupture Achilles rat model (AC), with unseeded aligned (A) and random (R) scaffolds as controls.^[^ ^117^ ^]^ After 4 weeks, the samples were harvested and further analyzed. Very mild scar formation was evident for the AC group (**Figure** **10**), compared to the A (mild) and R (prominent). AC group revealed an increase in tendon‐like tissue formation and collagen deposition. Histological quantification revealed a significantly improved score for the AC group compared to the other two, as well as for A compared to R. Immunohistochemical analysis revealed significantly higher expression of C206, an M2 antiinflammatory polarization marker, for both AC and A compared to R, and the opposite trend for iNOS M1 polarization marker, further highlighting the importance of the substrate topography in tendon repair.

However, both the random and aligned scaffold presented Young's modulus values in the range of 60–80 kPa, several orders of magnitude from the reported value for human Achilles tendon of 800 MPa.^[^ ^118^ ^]^ Though this study highlights the importance of an aligned material over a random material for clinical applications in tendon, it would require mechanical reinforcement before further testing.

Alternative materials have also been explored: Zhao et al.^[^ ^25^ ^]^ developed an ice templated scaffold utilizing human hair keratin, an abundant, biocompatible, nonimmunogenic, and biodegradable protein.^[^ ^119^ ^]^ The scaffolds presented a constant pore width of 5 µm when freezing at −80 °C; keratin concentration of 10 mg mL^−1^ resulted in a mostly fibrous pore structure with poor mechanical properties whereas concentrations above 20 mg mL^−1^ exhibited typical lamellar morphologies. Tensile testing revealed an increase in the ultimate tensile strength of the anisotropic scaffolds, from 0.2 to 1.2 MPa, when increasing the keratin concentration from 10 to 35 mg mL^−1^; though these values are still not in the range of the target application. Cell viability was analyzed by seeding scaffolds with human dermal fibroblasts, revealing no cytotoxicity, and interestingly, concentrations of 15 and 25 mg mL^−1^ exhibit increased viability compared to tissue culture plastic, indicating that keratin could promote cell adhesion. Fluorescence staining shows the cells align in the direction of the pores with their typical spindle‐like morphology.

Another pursued strategy for strengthening scaffolds is the fabrication of core‐shell type scaffolds, where one of the components is an ice templated material. This could yield a dense and strong component, providing adequate mechanical strength, with another spongy and aligned, to impart the right topographical cues for cell proliferation.

A collagen‐1,4‐butanediol diglycidyl ether composite made from a braided, dense core, surrounded by a spongy, aligned scaffold was produced and tested both in vitro with MSCs and in vivo,^[^ ^120^ ^]^ in an Achilles tendon rat model. The braided core achieved a maximum tensile strength of 6 MPa, in the range of application for supraspinatus tendon. Cell alignment along the direction of freezing and proliferation was verified in vitro for the shell component. Only the core component was tested in vitro, due to the complexity of the suture. This highlights one of the limitations of this strategy, as the interface between the components would need to be firmly secured prior to implantation to avoid excessive suturing during the operation. The tendons with the implanted braid exhibited well aligned collagen fibers when compared to the control group; however, signs of elongation and hypercellularity were present.

Similarly, a silk‐collagen scaffold was proposed for the tendon‐to‐bone interface, where osteogenic rather than tenogenic differentiation is preferable.^[^ ^121^ ^]^ The collagen was freeze casted around a silk braid in both random and aligned structures. In an in vitro study with rabbit MSCs, tenogenic gene expression increased for the aligned scaffold, including Col I and Col III genes, and cells presented a spindle‐like morphology. On the other hand, osteogenic markers rose for the isotropic scaffold, including BMP‐2 and RUNX‐2, with the cells presenting a more polygonal appearance.

A different alternative was proposed for a core–shell scaffold, where the core was produced from a unidirectionally frozen collagen‐GAG solution, and the shell an air dried membrane from the same source.^[^ ^122^ ^]^ In this case, the membrane was placed on the inside walls of the mold, and subsequently the solution was pipetted and allowed to hydrate the membrane before freezing. This could be an interesting approach to optimize the binding between layers. The tensile strength of the scaffold increased significantly with the thickness of the membrane; however, the composite still falls short for applications in tendon tissue. Interestingly, samples with and without membrane seeded with equine tendon cells exhibit no difference in cell number 7 and 14 days after seeding, indicating that the non‐porous membrane is not an impediment to cell proliferation.

Gouveia et al.^[^ ^123^ ^]^ studied a similarly produced unidirectional collagen core supplemented with proteoglycans and GAGs, surrounded by an electrospun PCL shell, for ligament tissue applications. An assessment of the incorporation of different proteoglycans and GAGs into the scaffolds core revealed that dermatan sulfate addition results in the greatest compressive strength, when compared to chondroitin sulfate, decorin and biglycan. This formulation also exhibits the highest metabolic activity after 14 days of seeding with human MSCs. Quantitative polymerase chain reaction (q‐PCR) analysis showed that the collagen‐decorin formulation has the highest type I/type III collagen ratio, which could be favorable for healing.^[^ ^124^ ^]^

Though considerable work has been done in the field of AIT for tendon tissue engineering, the scaffolds often fall short of the target tensile strength values for the application; however, important knowledge has been gained into the importance of an aligned substrate and substrate composition for the performance of tenocytes seeded on these materials. Further work should develop a focus on reinforcing these materials to reach the target mechanical properties, either by alternating the composition of the polymeric blend or combining AIT with other techniques, such as electrospinning and melt electrowriting.

## 5 Skeletal Muscle

Skeletal muscle, similarly to tendon, presents a hierarchical, aligned microstructure, formed by myofibers, blood vessels, nerves, and connective tissue. But unlike tendon, muscle is both highly vascularized and susceptible to electrical stimuli, which further difficult the design of appropriate biomaterials. The most common technique in use for the treatment of large muscle injuries consists of the application of autografts, however, the concerns with donor side morbidity and lack of donor source dictate a need for new alternatives.^[^ ^125^ ^]^

**Figure 11:**
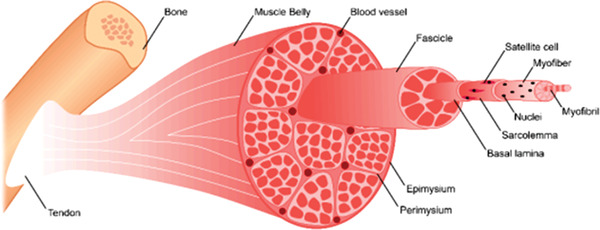
Schematic of the microstructure of skeletal muscle, where myofibers represent the structural units of the fascicles that make up the bulk of the tissue. Scaffolds designed for this application should support and promote the formation of myotubes in vitro and in vivo. Reproduced under an open access Creative Common CC BY license.^[^ ^129^ ^]^ Copyright 2020, the Authors. Published by MDPI.

Myofibers comprise the structural unit of muscle tissue (represented in **Figure** **11**). They are formed after the fusion of myoblasts into one elongated myotube, with multiple and centralized nuclei. In time, the nuclei move to the periphery and a myofiber is formed. Optimal biomaterials for muscle tissue should promote myotube formation, for which the topography of the substrate plays an important role. An aligned surface topography can allow the formation of parallel myotubes of up to two times greater length when compared to an anisotropic surface, as shown on aligned nanofibers.^[^ ^126^ ^]^ Similarly, when designing a scaffold for skeletal muscle tissue, the mechanical properties of native muscle should be closely matched, with reported values of stiffness for skeletal muscle in the range of 40–180 kPa;^[^ ^127^ ^]^ and the material should support the cyclical compression and relaxation movements that skeletal muscle undergoes naturally.^[^ ^128^ ^]^

In freeze casted scaffolds, pore size plays another important role. Myotubes with diameters up to 50 µm, comparable to native tissue, were observed on chitosan scaffolds with a median pore size of 110 µm after 14 days of seeding.^[^ ^127^ ^]^ An identical scaffold with 180 µm pore diameter also allowed for the successful formation of myotubes, however the diameter after 2 weeks was just 20 µm; and smaller pores of 28 um revealed no myotube formation. The stiffness values for these scaffolds ranged from 4 to 125 kPa, indicating they could be suitable for skeletal muscle applications.

On a collagen scaffold, it has been found that myoblast cells conform to the architecture of the scaffold regardless of size. Scaffolds with the smaller pore size of 98 µm, resulted in a slight increase in cell metabolic activity, and a significant increase in gene expression of myoblast determination (MyoD), indicating that the higher surface area can be beneficial for the cells through the increase of cell–cell signaling, key for myofiber formation.^[^ ^130^ ^]^

Electrical conductivity is characteristic of muscle tissue, allowing electrical signals to contract the tissue and transmit forces. Thus, mimetic biomaterials designed with an outlook on skeletal muscle tissue engineering should possess electroconductive properties. To this end, conductive polymers and carbon nanotubes have been studied as possible additives to a polymeric base in order to improve the materials conductive properties.^[^ ^17^, ^131^ ^]^

**Figure 12:**
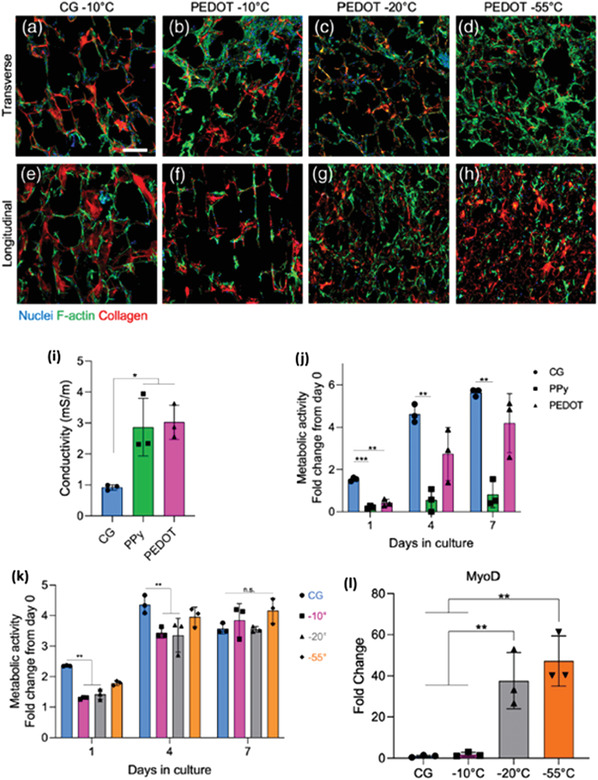
Collagen‐glycosaminoglycan (GAG) scaffold doped with electroconductive polymers for muscle tissue engineering. a–h) Fluorescence staining in both transversal and longitudinal directions for PEDOT containing scaffolds frozen at different temperatures. i) Conductivity of the collagen, poly(3,4‐ethylenedioxythiophene (PEDOT), and polypyrrole (PPy) loaded scaffolds. j) Metabolic activity of the collagen, PEDOT, and PPy scaffolds after 1, 4, and 7 days in culture. k) Metabolic activity of the PEDOT loaded scaffolds frozen at different temperatures. l) MyoD expression of the different PEDOT scaffolds. ^*^ *p* < 0.05, ^**^ *p* < 0.01, ^***^ *p* < 0.001. Reproduced under an open access Creative Common CC BY license.^[^ ^130^ ^]^ Copyright 2022, the Authors. Published by Wiley‐VCH GmbH.

PPy and poly(3,4‐ethylenedioxythiophene (PEDOT) have been investigated as candidates for improving the conductivity of a collagen‐GAG ice templated scaffold.^[^ ^130^ ^,132]^ PPy content between 0.2% and 0.5% is sufficient to significantly increase the conductivity of the material, with higher contents exhibiting cytotoxic behavior. Myoblast differentiation was found to improve in the presence of PPy, and the number of multinucleated myotubes increased; however, myogenic gene expression, measured by qPCR, was not found to improve when compared to the 0% PPy scaffold. PEDOT is an interesting alternative to Ppy, due to its lower toxicity to cells and increased conductivity. When embedded in the collagen‐GAG matrix, mouse myoblast proliferation was comparable for the PEDOT composite and the control (**Figure** **12**); while the PPy had significantly lower cell viability values; however, conductivity was similar for both PPY and PEDOT loaded scaffolds.

Carbon nanotubes have been incorporated as part of a polydopamin coating on aligned gelatin scaffolds in order to improve their conductivity.^[^ ^133^ ^]^ Utilizing a 7.5% gelatin content, the resulting scaffolds exhibit a smooth tubular structure similar to muscle tissue, with appropriate elastic behavior and stiffness value. An in vivo rat model showed that the incorporation of the nanotubes significantly increased the formation of new muscle fibers when compared to both uncoated and isotropic scaffolds. This could indicate that the synergistic effect of both an aligned topography and a conductive material is key for the success of these scaffolds.

## 6 Cartilage

**Figure 13:**
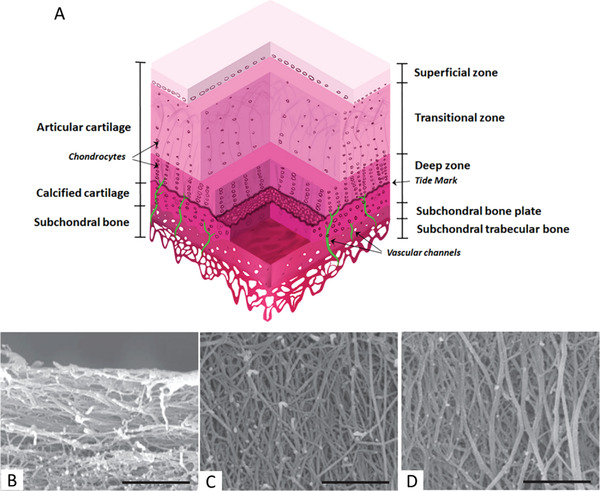
Cartilage microstructure, highlighting the three distinct zones and their morphology. A) Schematic representation of cartilage microstructure. The transitional zone has no preferred alignment, contrasting with the deep zone where the collagen fibers align vertically. B) Scanning electron microscopy (SEM) image of the superficial zone of pig articular cartilage, exhibiting horizontal alignment. C) SEM image of the transition zone of pig articular cartilage. No preferential orientation is evident. D) SEM image of the deep zone of pig articular cartilage, exhibiting vertical orientation. Scale bars: 2 µm. (A) Reproduced under an open access Creative Common CC BY license.^[^ ^140^ ^]^ Copyright 2020, the Authors. Published by Frontiers. (B–D) Reproduced under an open access Creative Common CC BY license.^[^ ^141^ ^]^ Copyright 2014, the Authors. Published by BioMed Central.

Cartilage is a rigid, avascular tissue present in the joints and many parts of the skeleton, facilitating joint articulation. Collagen (mainly type II) is an important part of its matrix, with a horizontal alignment toward the superficial zone, a transition zone with random orientation, and a vertical alignment in the area nearest to the bone.^[^ ^134^ ^]^ Water, GAGs, and hyaluronic acid are other important components. Microstructure and SEM images of the three zones are represented in **Figure** **13**.

As cartilage is a heterogeneous tissue, with distinct zones having different collagen alignments, mimicking its structure through tissue engineering is a complex task. The different zones possess different mechanical properties and chondrocyte densities, which must be accounted for in the scaffold design. Values for the mechanical properties of articular cartilage have been estimated in the range of 0,8–25 MPa for maximum tensile strength, and 0,24–0,85 MPa for compressive Young's modulus.^[^ ^135^ ^]^ Furthermore, a wide range of pore sizes has been found to be required for optimal cell distribution throughout the different zones of the material.^[^ ^136^ ^]^

Many polymers, including PCL,^[^ ^8^ ^]^ collagen,^[^ ^137^ ^]^ silk,^[^ ^138^ ^]^ chitosan,^[^ ^139^ ^]^ and cellulose have been used to produce freeze casted materials, and achieved mechanical properties similar to cartilage. However, successful approaches to the design of a biomaterial for cartilage could require additional complexity to mimic the multilayer structure.

**Figure 14:**
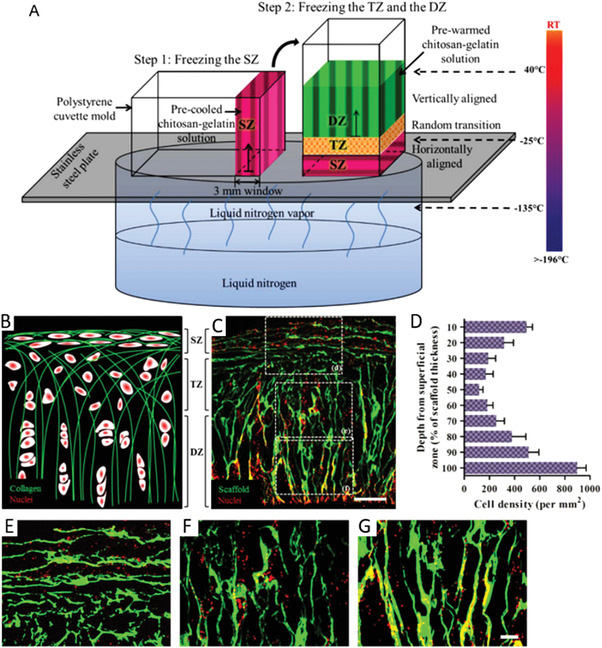
Chitosan–gelatin bilayer scaffold produced with sequential freezing. a) Schematic representation of the bidirectional freezing method. b) Schematic representation of the collagen fiber alignment and cell infiltration. SZ: Superficial zone; TZ: Transition zone; DZ: Deep Zone. c) Fluorescence staining image of the seeded scaffold. d) Cell densities in the different zones of the scaffold. e–g) Fluorescence staining images of the SZ, TZ, and DZ, respectively. Reproduced with permission.^[^ ^26^ ^]^ Copyright 2015 Elsevier Ltd.

In this regard, bilayered scaffolds can both mimic the cartilage and bone layers, introducing both micro and macroporosity for the transport of nutrients and cells. An interesting approach to recreate this structure was presented by sequentially freezing, first a layer of chitosan‐gelatin solution in one direction, followed by a second layer frozen at 90° with respect to the first one, as illustrated in **Figure** **14**.^[^ ^26^ ^]^ In this way, a scaffold can possess an architecture similar to native cartilage, with horizontally aligned polymer in the surface, followed by vertically aligned pores in the rest of the material. These multizonal scaffolds presented similar compressive behavior to unidirectional scaffolds although improved flexibility, with values similar to the properties of pericellular matrix, favorable for chondrogenesis.^[^ ^142^ ^]^ Cell infiltration into the deep zone of the scaffolds was successfully demonstrated via fluorescence microscopy.

A combination of extrusion and unidirectional freeze casting techniques was studied to mimic the bone and cartilages layers using TCP and collagen.^[^ ^137^ ^]^ By extruding a TCP slurry into rods, sintering, and placing them on top of a collagen solution followed by freeze casting, both micro and macropores were achieved. This allows for the delivery of nutrients and cells, while still retaining the appropriate mechanical properties. An excellent repair of the patellar cartilage was observed in rabbit in vivo model for scaffolds with channels of 270 µm, with a bone regeneration ratio of over 70% after 12 weeks. Both soft tissue and new bone formation could be appreciated via histological staining. Larger channels were found to delay differentiation of the regenerated new tissue into bone; whereas with smaller channels bone regenerated well but cell density was lower than for the 270 µm version.

Cartilage regeneration remains a clinical challenge, due to its lack of vascularization and complexity of the extracellular matrix. AIT provides a promising platform for aiding cartilage healing and repair, however the complexity of the tissue could require the use of a combined technique, as described in this section, to achieve a successful biomaterial.

## 7 Conclusions and Outlook

Anisotropic structures are found in tissues throughout the musculoskeletal system. Mimicking these morphologies is key to producing biomaterials with an outlook on regenerative medicine and tissue engineering. Macroporous aligned scaffolds have been linked to improved mechanical properties, cell alignment, tenogenic differentiation potential, and improved immune response when compared to isotropic materials.

AIT is a low‐cost, versatile, and reproducible technique that requires little equipment to produce oriented macroporous structures. This review discussed some of the many possibilities brought forth by this process, capable to produce biomaterials with an array of pore sizes in the 1–400 µm range, utilizing ceramics, metals, and polymers. The optimization of the materials morphology can be accomplished by modifying numerous parameters, including particle size, freezing conditions, and slurry composition. The highlighted research showed that by tuning the scaffolds pore structure and mechanical properties, successful biomaterials can be produced for applications including bone, tendon, cartilage, and ligament.

In the versatility of the technique also lie some of its challenges: With so many parameters to optimize, the prediction of the final material characteristics is complex and requires further study. Moreover, the lack of commercial equipment for the production of ice templated biomaterials could difficult the reproducibility of the technique, given the difficulties to precisely control for parameters such as temperature gradient in homemade devices. As the interest in AIT grows, there will be a need for the industrial sector to fill the gap and supply researchers with standardized equipment capable of strict parameter control, which will in turn help advance knowledge.

Emerging technologies, including machine learning and artificial intelligence, could provide tools for researchers in predicting some of the final materials properties.^[^ ^24^ ^]^ Recently, a machine learning model was capable of accurately predict the degradation behavior of a gelatin porous scaffold;^[^ ^143^ ^]^ indicating that with further work this technique could prove useful as a predictive tool.

For polymeric materials produced with the AIT technique, additional work is required to further understand the influence of cross‐linking conditions, as it can have significant effects on morphology, degradation kinetics, and biophysical properties. Furthermore, gelation time could be identified as an under‐researched parameter that could prove to be influential in the final properties of the material.

In the case of ceramics, many opportunities exist to optimize the degradation and load bearing behavior of AIT developed biomaterials; further investigations are expected to progress toward improving their biocompatibility and cellular viability.

When looking at each of the individual tissues reviewed, the bulk of the research has focused on applications for bone. Interestingly, little attention has been given to the development of bilayer or core–shell scaffolds using AIT for bone, as opposed to tendon and cartilage, and it could prove an interesting development considering the two distinct microstructures of trabecular and cortical bone. Generally, scaffolds performed better under in vitro and in vivo testing when a bioactive component was incorporated, indicating that good biocompatibility is not enough for biomaterials intended for bone, and bioactivity should also be minded in the scaffolds design.

Furthermore, more work could be focused on developing a cell‐loaded aligned scaffold as a regenerative therapy in an in vivo model, particularly in tissues such as tendon and cartilage, which suffer from low vascularization and slow healing.

Finally, research work is anticipated to continue in the direction of understanding the complex relationships between pore morphology, cellular interactions, mechanical properties, material composition, and drug and protein delivery for freeze casted scaffolds, aiming at developing superior AIT biomaterials that are suitable to be translated to clinical applications in the musculoskeletal system.

## Conflict of Interest

The authors declare no conflicts of interest.
